# Morbimortalité périnatale dans les grossesses gémellaires dans une maternité marocaine de niveau 3

**DOI:** 10.11604/pamj.2016.23.80.8789

**Published:** 2016-03-10

**Authors:** Mohamed El-Mahdi Boubkraoui, Hassan Aguenaou, Mustapha Mrabet, Amina Barkat

**Affiliations:** 1 Centre National de Référence en Néonatologie et Nutrition, Hôpital d'enfants de Rabat, Maroc; 2 Équipe de Recherche sur la Santé et la Nutrition de la Mère et de l'Enfant, Faculté de Médecine et de Pharmacie de Rabat, université Mohammed V de Rabat, Maroc; 3 Unité Mixte de Recherche en Nutrition et Alimentation URAC 39, Université Ibn Tofail-CNESTEN RDC-Nutrition AFRA/AIEA, Rabat, Maroc; 4 Département de Santé Publique, Faculté de Médecine et de Pharmacie de Rabat, Université Mohammed V de Rabat, Maroc

**Keywords:** Grossesse gémellaire, morbidité périnatale, mortalité périnatale, Maroc, Twin pregnancy, perinatal morbidity, perinatal mortality, Morocco

## Abstract

**Introduction:**

Les grossesses gémellaires sont associées à un risque de morbimortalité périnatale plus élevé que les grossesses simples. Le but de cette étude était d’évaluer la morbimortalité périnatale dans les grossesses gémellaires dans une maternité marocaine de niveau 3.

**Méthodes:**

Il s'agit d'une étude transversale comparative de la morbimortalité périnatale des nouveau-nés issus de grossesses gémellaires versus grossesses simples ayant accouché à la maternité Souissi de Rabat du 1 janvier au 28 février 2014.

**Résultats:**

Il y a eu 3297 naissances issues de 65 grossesses gémellaires et 3167 grossesses simples. Les grossesses gémellaires étaient associées à des taux plus élevés de prééclampsie et d’éclampsie (P = 0,046), de HELLP syndrome (P= 0,030), de rupture prématurée des membranes (P < 0,001), de présentation dystocique (P < 0,001), de prématurité (P < 0,001), d'hypotrophie chez les nouveau-nés à terme (P < 0,001), de détresse respiratoire néonatale (P < 0,001), de malformations congénitales (P = 0,015), d'hospitalisation en période néonatale (P = 0,001) et de mortalité périnatale (P = 0,001) par rapport aux grossesses simples. Les jumeaux monochoriaux présentaient des taux plus élevés d'hypotrophie en cas de grossesse menée à terme (P = 0,016) et de mortalité périnatale (P = 0,017) par rapport aux jumeaux bichoriaux.

**Conclusion:**

Les grossesses gémellaires étaient à risque plus élevé de morbimortalité périnatale par rapport aux grossesses simples en exposant notamment à la prématurité. Les grossesses gémellaires monochoriales étaient plus à risque en exposant notamment à l'hypotrophie chez les nouveau-nés à terme.

## Abstract (en)

**Introduction:**

Twin pregnancies are associated with a higher rate of perinatal morbidity and mortality than singleton pregnancies. The aim of the present study was to evaluate perinatal morbidity and mortality in twin pregnancies in a Moroccan level-3 maternity ward.

**Methods:**

This is a comparative cross-sectional study of perinatal morbidity and mortality rates in newborn infants in twin pregnancies versus singleton pregnancies among women who gave birth at Souissi Maternity Hospital in Rabat from January 1 to February 28, 2014.

**Results:**

There were 3297 births and, of these, 65 in twin pregnancies and 3167 in singleton pregnancies. Twin pregnancies were associated with higher rates of preeclampsia and eclampsia (P = 0.046), HELLP syndrome (P = 0.030), premature rupture of membranes (P < 0.001), malpresentation (P < 0.001), prematurity (P < 0.001), low birth weight in fullterm neonates (P < 0.001), respiratory distress at birth (P < 0.001), congenital malformations (P = 0.015), hospitalization in the neonatal period (P = 0.001), and perinatal mortality (P = 0.001) than singleton pregnancies. Monochorionic twins showed higher rates of low birth weight in fullterm pregnancies (P = 0.016) and of perinatal mortality (P = 0.017) than dichorionic twins.

**Conclusion:**

Twin pregnancies showed higher risk of perinatal morbidity and mortality than singleton pregnancies and were more exposed to prematurity. Monochorionic twin pregnancies showed a higher risk because of the significant exposure to low birth weight in fullterm babies.

## Introduction

La grossesse gémellaire est définie comme le développement simultané de deux embryons dans l'utérus. Elle est considérée comme une grossesse à risque élevé et justifie toujours une attention particulière. La morbimortalité périnatale est trois à sept fois plus élevée chez les jumeaux comparativement aux singletons en raison notamment d'une fréquence plus élevée de la prématurité, de l'hypotrophie et des accouchements dystociques [1]. De plus, les jumeaux issus de grossesses monochoriales sont plus à risque que les jumeaux issus de grossesses bichoriales du fait du risque de syndrome transfuseur-transfusé qui survient dans environ 10% des grossesses monochoriales [2]. L'objectif de cette étude était d’évaluer la morbimortalité périnatale et les facteurs qui y sont associés dans les grossesses gémellaires en comparaison avec les grossesses simples dans une maternité marocaine de niveau 3. À notre connaissance, ceci est la première étude réalisée sur ce sujet au Maroc.

## Méthodes

### Type et lieu de l’étude

Nous avons mené cette étude sur une période de deux mois entre le 1 janvier 2014 et le 28 février 2014 à la maternité Soussi qui est une maternité de niveau 3 du centre hospitalier universitaire Ibn Sina de Rabat. Il s'agit d'une étude transversale comparative de la morbimortalité périnatale des nouveau-nés issus de grossesses gémellaires versus grossesses simples durant la période de l’étude.

### Critères d'inclusion et d'exclusion

Les critères d'inclusion des patients se sont basés sur la définition de l'OMS de la viabilité du nouveau-né qui est un poids à la naissance d'au moins 500 g et un âge gestationnel d'au moins 22 semaines. Les nouveau-nés pesant moins de 500 g ou qui avaient un âge gestationnel inférieur à 22 semaines ont été exclus. Il n'y avait pas d'autres critères d'exclusion.

### Collecte des données

Les données suivantes ont été recueillies prospectivement pour chaque naissance par les résidents en pédiatrie de garde à la maternité Souissi: âge maternel, poids et taille maternels, maladie maternelle chronique, antécédents obstétricaux, mode de conception, suivi de la grossesse, complications de la grossesse, corticothérapie anténatale, présentation du fœtus, mode d'accouchement, chorionicité en cas de grossesse multiple, date de naissance, sexe et poids du nouveau-né, âge gestationnel, mort fœtale *in utero*, score d'Apgar, survenue d'une détresse respiratoire néonatale, anomalies à l'examen physique du nouveau-né, hospitalisation en période néonatale, décès en période néonatale précoce et cause.

### Considérations éthiques

Cette étude a été approuvée par le comité d’éthique de la faculté de médecine et de pharmacie de Rabat. Un consentement éclairé a été obtenu de chaque participante. La collecte des données était anonyme en utilisant le numéro de dossier des parturientes.

### Définition des termes

La grossesse était considérée comme bien suivie lorsqu'il y avait eu trois consultations prénatales avec trois échographies obstétricales. La chorionicité a été déterminée par l’échographie obstétricale ou par l'examen du placenta à la naissance des grossesses multiples. L'obésité maternelle a été définie par un indice de masse corporelle supérieur à 29,9 kg/m^2^ [3]. La césarienne était programmée si la décision d'effectuer l'opération avait été prise avant le début du travail et après la préparation préopératoire à un moment pré-arrangé pendant les heures ouvrables pour garantir la meilleure prise en charge possible obstétricale et néonatale, même lorsque le travail a commencé avant l'intervention. Les autres césariennes ont été considérées comme étant des césariennes urgentes. La parité était le nombre de grossesses précédentes se terminant après 20 semaines de gestation y compris en cas de mortinatalité. L'intervalle entre la naissance des jumeaux a été considéré comme long lorsqu'il dépassait 30 minutes [4]. La mortalité néonatale précoce inclut tous les décès survenus dans les sept premiers jours de vie. La mortalité périnatale a été définie comme la somme de tous les mort-nés et des décès néonatals précoces. La prématurité a été définie comme une naissance survenant avant 37 semaines de gestation. La postmaturité a été définie comme une gestation de 42 semaines ou plus. Un faible poids de naissance a été défini comme un poids de naissance inférieur au dixième percentile des courbes de référence. La macrosomie a été définie comme un poids de naissance supérieur au quatre-vingt-dixième percentile des courbes de référence [5]. L’âge gestationnel a été calculé en utilisant la date des dernières règles de la mère si elle était connue ou a été estimé par l’échographie obstétricale ou par le score de Dubowitz à la naissance [6]. L'asphyxie périnatale a été définie comme un score d'Apgar inférieur à sept à cinq minutes de vie [7]. Une infection maternofœtale a été soupçonnée en cas de chorioamniotite ou de rupture prématurée de 12 heures ou plus des membranes. La discordance du poids de naissance des jumeaux a été calculée en utilisant la formule suivante: ([poids de naissance du plus grand jumeau] - [poids de naissance du plus petit jumeau]) ÷ [poids de naissance du plus grand jumeau] × 100 [8]. Le poids de naissance des jumeaux était considéré comme concordant si la différence de poids des jumeaux ne dépassait pas 5%. La discordance du poids de naissance des jumeaux était considérée comme modérée si elle était comprise entre 5 et 25% et elle était considérée comme importante si elle dépassait 25% [9]. Un syndrome transfuseur-transfusé a été suspecté en cas de discordance importante du poids de naissance de jumeaux du même sexe issus d'une grossesse monochoriale à défaut d'un diagnostic échographique anténatal sur les critères internationalement acceptés avec une séquence hydramnios polyurique chez le receveur et oligoanamnios anurique chez le donneur [10].

### Étude statistique

Les caractéristiques maternelles, obstétricales, du nouveau-né et de l'issue périnatale ont été analysées. Les données ont été exprimées en pourcentage, moyenne et écart-type. Le test de khi-deux de Pearson a été utilisé pour comparer les variables qualitatives et le test de Student a été utilisé pour comparer les variables quantitatives. Une valeur P inférieure à 0,05 a été considérée comme statistiquement significative.

## Résultats

Il y a eu durant la période de l’étude 3297 naissances issues de 65 grossesses gémellaires et 3167 grossesses simples. Il y avait 1619 nouveau-nés de sexe masculin (49,11%) et 1678 nouveau-nés de sexe féminin (50,89%), soit un sex-ratio mâle-femelle de 0,96. Pour les 3274 naissances vivantes recensées (99,30%), il y a eu 20 mort-nés (60% étaient frais et 40% étaient macérés) et 3 cas de mortalité néonatale précoce, soit un taux de mortalité périnatale de 7 pour 1000 naissances. Il y avait au total 770 cas de morbidité périnatale (23,35%) toutes grossesses confondues. Toutes les conceptions étaient spontanées. La prévalence des grossesses gémellaires était de 2,01%. Il n'y a eu aucune grossesse triple ni autre grossesse de haut rang ni jumeaux siamois durant la période de l’étude. L’âge maternel variait de 16 à 51 ans. Il était en moyenne de 29,71 ± 6,35 ans dans le groupe des grossesses gémellaires et en moyenne de 28,66 ± 6,25 ans dans le groupe des grossesses simples. Les mères étaient âgées de plus de 40 ans dans 9,23% des cas pour les grossesses gémellaires et dans 2,24% des cas pour les grossesses simples et cette différence était statistiquement significative (P < 0,001). Les mères étaient obèses dans respectivement 38,46% et 37,16% des cas dans le groupe des grossesses gémellaires et le groupe des grossesses simples. Un antécédent de grossesse gémellaire était plus fréquent en cas de grossesse gémellaire (P = 0,003). Les mères étaient primipares dans 41,54% des cas pour les grossesses gémellaires et dans 34,99% des cas dans les grossesses simples. La grossesse était bien suivie dans respectivement 86,15% et 83,80% des cas dans le groupe des grossesses gémellaires et le groupe des grossesses simples. La grossesse gémellaire était méconnue avant l'accouchement dans 13,85% des cas. La fréquence de la prééclampsie et de l’éclampsie (P = 0,046), du HELLP syndrome (P = 0,030) et de la rupture prématurée des membranes (P < 0,001) était plus élevée en cas de grossesse gémellaire. Les accouchements par voie basse spontanée étaient plus fréquents en cas de grossesse simple (P = 0,015) et le recours à l'accouchement par césarienne en urgence était plus fréquent en cas de grossesse gémellaire (P = 0,002). Une corticothérapie anténatale en cas de prématurité inférieure à 35 semaines a été donnée dans 66,67% des cas aussi bien dans les grossesses gémellaires que dans les grossesses simples.

Le poids moyen de naissance des jumeaux (2467 ± 567 g) était plus faible que celui des singletons (3201 ± 593 g) et cet écart était significatif (P < 0,001). Les jumeaux étaient de sexe différent dans 27,69% des cas, il y a eu une paire de jumeaux de sexe masculin dans 36,92% des cas et une paire de jumeaux de sexe féminin dans 35,39% des cas. Les poids des jumeaux d'une même paire étaient concordants dans 33,85% des cas. La discordance entre le poids d'une paire de jumeaux était modérée dans 56,92% des cas et importante dans 9,23% des cas. L’âge gestationnel moyen des jumeaux était de 37,08 ± 2,56 semaines contre 38,12 ± 2,27 semaines pour les singletons et cette différence était significative (P < 0,001). Il a été retrouvé de manière significative (P < 0,001) qu'une infection maternofœtale était plus fréquemment suspectée chez les jumeaux (30,77%) que chez les singletons (12,79%). Une présentation de siège ou une autre présentation dystocique était plus fréquente (P < 0,001) chez les jumeaux (28,46%) que chez les singletons (2,53%). La proportion des combinaisons de présentations des jumeaux étaient comme suit: céphalique-céphalique (58,46%), céphalique-siège (13,85%), siège-céphalique (13,85%), siège-siège (10,77%), céphalique-transverse (1,54%), siège-transverse (1,54%). Les jumeaux à terme étaient plus souvent hypotrophes (30,00%) que les singletons (1,36%) et cette différence était significative (P < 0,001). Le taux des jumeaux prématurés (27,69%) était plus élevé (P < 0,001) que celui des singletons prématurés (0,28%). Une détresse respiratoire néonatale était survenue plus fréquemment (P < 0,001) chez les jumeaux (10,00%) que chez les singletons (1,80%). Le taux de malformations congénitales était plus élevé (P = 0,015) chez les jumeaux (2,31%) que chez les singletons (0,57%). Les traumatismes obstétricaux étaient plus élevés (P = 0,030) chez les jumeaux (0,77%) par rapport aux singletons (0,09%). Les hospitalisations en période néonatale étaient plus fréquentes (P = 0,001) chez les jumeaux (36,15%) par rapport aux singletons (22,83%). La fréquence des mort-nés était plus élevée (P = 0,011) chez les jumeaux (2,31%) que chez les singletons (0,54%). Le taux de mortalité néonatale précoce était également plus élevé (P = 0,009) chez les jumeaux (0,77%) que chez les singletons (0,06%). La mortalité périnatale était ainsi de 30,8 pour mille naissances chez les jumeaux et de 6 pour mille naissances chez les singletons avec une différence significative (P < 0,001). Les caractéristiques maternelles, obstétricales, des nouveau-nés et de l'issue périnatale sont énumérées dans le Tableau 1.

**Tableau 1:** Caractéristiques maternelles, obstétricales, des nouveau-nés et de l'issue périnatale dans les grossesses multiples versus grossesses simples

Caractéristiques maternelles et obstétricales dans les grossesses multiples versus grossesses simples	Grossesses gémellaires (n = 65)	Grossesses simples (n = 3167)	*P*
Caractéristiques maternelles			
Âge maternel moyen (années)	29,71 ± 6,35	28,66 ± 6,25	0,187
Âge maternel supérieur à 40 ans	6 (9,23)	71 (2,24)	< 0,001
Âge maternel inférieur à 18 ans	0 (0)	68 (2,15)	0,232
Indice de masse corporelle	28,8 ± 4,6	28,2 ± 5,4	0,300
Obésité	25 (38,46)	1177 (37,16)	0,594
Antécédent de grossesse gémellaire	3 (4,62)	29 (0,92)	0,003
Antécédent d'avortement spontané	1 (1,54)	234 (7,39)	0,072
Mère primipare	27 (41,54)	1108 (34,99)	0,273
Diabète	0 (0)	14 (0,44)	0,591
Hypertension artérielle chronique	0 (0)	6 (0,19)	0,667
Caractéristiques obstétricales			
Bon suivi de la grossesse	56 (86,15)	2654 (83,80)	0,610
Méconnaissance de la grossesse gémellaire	9 (13,85)	-	-
Diabète gestationnel	0 (0)	7 (0,22)	0,592
Hypertension artérielle gravidique	3 (4,62)	37 (1,17)	0,245
Prééclampsie et éclampsie	2 (3,08)	12 (0,38)	0,046
HELLP syndrome	1 (1,54)	3 (0,09)	0,030
Hématome rétroplacentaire	0 (0)	3 (0,09)	0,726
Rupture prématurée des membranes	19 (29,23)	222 (7,01)	< 0,001
Chorioamniotite	1 (1,54)	183 (5,78)	0,144
Accouchement par voie basse spontané	40 (61,54)	2371 (74,88)	0,015
Accouchement par voie basse instrumentalisé	5 (7,69)	248 (7,83)	0,967
Accouchement par césarienne programmée	3 (4,62)	136 (4,29)	0,899
Accouchement par césarienne en urgence	17 (26,15)	412 (13,01)	0,002
Accouchement par voie basse-césarienne en urgence	0 (0)	-	-
Intervalle entre l'accouchement des jumeaux (minutes)	6,28 ± 7,37	-	-
Intervalle long entre l'accouchement des jumeaux	1 (1,54)	-	-
Grossesses gémellaires monochoriales	16 (24,62)	-	-
Grossesses gémellaires bichoriales	49 (75,38)	-	-
Suspicion de syndrome transfuseur-transfusé	3 (4,61)	-	-
**Caractéristiques des nouveau-nés et de l'issue périnatale dans les grossesses multiples versus grossesses simples**	**Jumeaux** **(n = 130)**	**Singletons** **(n = 3167)**	***P***
Caractéristiques des nouveau-nés			
Sexe masculin	66 (50,77)	1559 (49,23)	0,730
Sexe différent avec l'autre jumeau	36 (27,69)	-	-
Poids moyen à la naissance (grammes)	2467 ± 567	3201 ± 593	< 0,001
Discordance modérée du poids de naissance avec l'autre jumeau	74 (56,92)	-	-
Discordance importante du poids de naissance avec l'autre jumeau	12 (9,23)	-	-
Âge gestationnel moyen (semaines)	37,08 ± 2,56	38,12 ± 2,27	< 0,001
Caractéristiques de l'issue périnatale			
Suspicion d'infection maternofœtale	40 (30,77)	405 (12,79)	< 0,001
Présentation de siège ou autre présentation dystocique	37 (28,46)	80 (2,53)	< 0,001
Exposition prénatale à une infection maternelle	1 (0,77)	6 (0,19)	0,159
Macrosomie à terme	0 (0)	54 (1,71)	0,133
Hypotrophie à terme	39 (30,00)	43 (1,36)	< 0,001
Post-terme	0 (0)	33 (1,04)	0,242
Prématurité	36 (27,69)	9 (0,28)	< 0,001
Asphyxie périnatale	1 (0,77)	130 (4,10)	0,056
Détresse respiratoire néonatale	13 (10,00)	57 (1,80)	< 0,001
Malformations congénitales	3 (2,31)	18 (0,57)	0,015
Traumatisme obstétrical	1 (0,77)	3 (0,09)	0,030
Hospitalisation en période néonatale	46 (35,38)	723 (22,83)	0,001
Mort-né	3 (2,31)	17 (0,54)	0,011
Mortalité néonatale précoce	1 (0,77)	2 (0,06)	0,009
Mortalité périnatale	4 (3,08)	19 (0,60)	0,001

*Les valeurs sont exprimées en moyenne ± écart-type ou en nombre (pourcentage)

Le poids à la naissance des deuxièmes jumeaux était en moyenne plus faible que celui des premiers jumeaux. De même, il y avait dans le groupe des deuxièmes jumeaux une fréquence plus élevée de présentations dystociques, de détresses respiratoires néonatales et d'hospitalisations en période néonatale. Ces différences n’étaient cependant pas statistiquement significatives. L'accouchement des grossesses gémellaires a eu lieu par voie basse dans 69,23% des cas et par césarienne dans 30,77% des cas. L'accouchement par voie basse était spontané dans 88,89% des cas et instrumentalisé dans 11,11% des cas. Les césariennes étaient programmées dans 15,00% des cas et urgentes dans 85,00% des cas. Les principales indications de la césarienne en cas de grossesse gémellaire étaient un utérus cicatriciel (20,00%), une rupture prématurée des membranes (20,00%), une présentation de siège du premier jumeau (15,00%), une prééclampsie (15,00%) et une hypertension artérielle gravidique (15,00%). L'accouchement des grossesses gémellaires par césarienne était associé à la survenue plus fréquente de détresse respiratoire néonatale et à un taux d'hospitalisations en période néonatale plus élevé par rapport à l'accouchement par voie basse sans que cette différence ne soit statistiquement significative. Les jumeaux prématurés sont nés plus fréquemment par voie basse que par césarienne et cette différence était significative (P = 0,031). La grossesse était monochoriale dans 24,62% des cas et bichoriale dans 75,38% des cas. Les jumeaux monochoriaux avaient un poids moyen à la naissance plus faible (P = 0,002) et avaient un risque accru de croissance discordante (P < 0,001), ils présentaient plus fréquemment une hypotrophie pour les nouveau-nés à terme (P = 0,016) et avaient une mortalité périnatale plus élevée (P = 0,017). L’âge gestationnel moyen des jumeaux monochoriaux était plus faible (36,25 semaines) que celui des jumeaux bichoriaux (37,35 semaines) sans que cela ne soit statistiquement significatif. Un syndrome transfuseur-transfusé a été suspecté dans le cas de trois paires de jumeaux monochoriaux chez qui le donneur présentait une croissance très retardée. Les nouveau-nés issus de grossesses gémellaires étaient prématurés dans 27,69% des cas et à terme dans 72,31% des cas. Les jumeaux prématurés ont présenté plus fréquemment une détresse respiratoire néonatale (P < 0,001) et ont plus souvent nécessité une hospitalisation en période néonatale (P = 0,010) que les jumeaux à terme. L'absence de suivi de la grossesse a été associée à une issue périnatale moins favorable chez les jumeaux sans que cette différence ne soit significative. L'issue périnatale des jumeaux en présentation dystocique était moins favorable, mais n’était pas significativement différente de celle des jumeaux en présentation céphalique. Les caractéristiques de l'issue périnatale des jumeaux sont présentées dans le Tableau 2.

**Tableau 2:** Caractéristiques de l'issue périnatale des jumeaux

Caractéristiques de l'issue périnatale des premiers jumeaux versus deuxièmes jumeaux	Premiers jumeaux (n = 65)	Deuxièmes jumeaux (n = 65)	*P*
Poids moyen à la naissance (grammes)	2497 ± 553	2438 ± 557	0,546
Présentation de siège ou autre présentation dystocique	17 (26,15)	20 (30,77)	0,560
Hypotrophie à terme	18 (27,69)	21 (32,31)	0,566
Détresse respiratoire néonatale	6 (9,23)	7 (10,77)	0,770
Hospitalisation en période néonatale	22 (33,85)	25 (38,46)	0,584
Mortalité périnatale	2 (3,08)	2 (3,08)	1,000
**Caractéristiques de l'issue périnatale des jumeaux nés par voie basse versus césarienne**	**Jumeaux nés par voie basse** **(n = 90)**	**Jumeaux nés par césarienne** **(n = 40)**	***P***
Poids moyen à la naissance (grammes)	2462 ± 550	2479 ± 615	0,883
Présentation de siège ou autre présentation dystocique	23 (25,56)	15 (37,50)	0,167
Hypotrophie à terme	26 (28,89)	13 (32,50)	0,678
Prématurité	30 (33,33)	6 (15,00)	0,031
Détresse respiratoire néonatale	3 (3,33)	7 (17,50)	0,005
Hospitalisation en période néonatale	28 (31,11)	18 (45,00)	0,126
Mortalité périnatale	3 (3,33)	1 (2,50)	0,800
**Caractéristiques de l'issue périnatale des jumeaux monochoriaux versus jumeaux bichoriaux**	**Jumeaux monochoriaux** **(n = 32)**	**Jumeaux bichoriaux** **(n = 98)**	***P***
Poids moyen à la naissance (grammes)	2167 ± 661	2565 ± 501	0,002
Discordance du poids de naissance avec l'autre jumeau	30 (93,75)	56 (57,14)	< 0,001
Âge gestationnel moyen (semaines)	36,25 ± 2,86	37,35 ± 2,43	0,005
Présentation de siège ou autre présentation dystocique	12 (37,50)	25 (25,51)	0,192
Hypotrophie à terme	15 (46,88)	24 (24,49)	0,016
Prématurité	10 (31,25)	26 (26,53)	0,640
Détresse respiratoire néonatale	3 (9,38)	10 (10,20)	0,892
Hospitalisation en période néonatale	10 (31,25)	36 (37,76)	0,573
Mortalité périnatale	3 (9,38)	1 (2,04)	0,017
**Caractéristiques de l'issue périnatale des jumeaux prématurés versus jumeaux à terme**	**Jumeaux prématurés** **(n = 36)**	**Jumeaux à terme** **(n = 94)**	***P***
Suspicion d'infection maternofœtale	12 (33,33)	28 (29,79)	0,695
Présentation de siège ou autre présentation dystocique	9 (25,00)	28 (29,79)	0,588
Détresse respiratoire néonatale	9 (25,00)	4 (4,26)	< 0,001
Hospitalisation en période néonatale	19 (52,78)	27 (28,72)	0,010
Mortalité périnatale	2 (5,56)	2 (2,13)	0,311
**Caractéristiques de l'issue périnatale des jumeaux issus des grossesses bien suivies versus grossesses mal suivies**	**Jumeaux issus des grossesses bien suivies** **(n = 26)**	**Jumeaux issus des grossesses mal suivies** **(n = 104)**	***P***
Suspicion d'infection maternofœtale	12 (46,15)	28 (26,92)	0,057
Présentation de siège ou autre présentation dystocique	9 (34,62)	28 (26,92)	0,437
Accouchement par césarienne en urgence	8 (30,77)	26 (25,00)	0,549
Hypotrophie à terme	8 (30,77)	31 (29,81)	0,924
Prématurité	6 (23,08)	30 (28,85)	0,557
Détresse respiratoire néonatale	2 (7,69)	11 (10,58)	0,661
Hospitalisation en période néonatale	12 (46,15)	34 (32,69)	0,199
Mortalité périnatale	1 (3,85)	3 (2,88)	0,800
**Caractéristiques de l'issue périnatale des jumeaux en présentation dystocique versus présentation céphalique**	**Jumeaux en présentation dystocique** **(n = 37)**	**Jumeaux en présentation céphalique** **(n = 93)**	***P***
Suspicion d'infection maternofœtale	10 (27,03)	30 (32,26)	0,560
Accouchement par césarienne en urgence	13 (35,14)	21 (22,58)	0,142
Hypotrophie à terme	11 (29,73)	28 (30,11)	0,966
Prématurité	11 (29,73)	25 (26,88)	0,743
Détresse respiratoire néonatale	4 (10,81)	9 (9,68)	0,846
Hospitalisation en période néonatale	11 (29,73)	35 (37,63)	0,395
Mortalité périnatale	1 (2,70)	3 (3,23)	0,876

*Les valeurs sont exprimées en moyenne ± écart-type ou en nombre (pourcentage)

## Discussion

La prévalence des grossesses gémellaires était modérément élevée dans notre étude (2,01%). Toutes les grossesses gémellaires recensées ont pourtant été conçues naturellement. Cette prévalence de grossesses gémellaires observée serait plutôt liée au fait que la maternité Souissi draine des patientes d'un large bassin autour de la ville de Rabat. La proportion des mères âgées de plus de 40 ans ou qui avaient un antécédent de grossesse gémellaire était significativement plus élevée dans le groupe des grossesses gémellaires par rapport au groupe des grossesses simples. Ceci est en accord avec les résultats de l’étude de Bortolus *et al*. qui ont démontré que l’âge maternel et les facteurs héréditaires étaient les déterminants les mieux définis favorisant les grossesses multiples spontanées [11]. Les complications des grossesses observées dans notre étude étaient significativement plus fréquentes en cas de grossesse gémellaire et il s'agissait le plus souvent d'une rupture prématurée des membranes suivie par les troubles hypertensifs. Une fréquence élevée des ces complications lors des grossesses gémellaires a été rapportée dans diverses études [12–14]. Le taux d'accouchements par voie basse spontanée (61,54%) était significativement moins élevé dans notre étude en cas de grossesse gémellaire par rapport aux grossesses simples, mais ce taux restait plus élevé que le taux retrouvé par Bhavana *et al*. (46,67%) et Persad *et al*. (56,73%) [15, 16]. De même le taux d'accouchements par césarienne était significativement plus élevé (26,15%), mais avec un taux moins élevé que celui rapporté par Bhavana *et al*. (53,33%) et Persad *et al*. (38,92%). Obiechina *et al*. ont montré dans leur étude que le recours à la césarienne était trois fois plus fréquent en cas de grossesse gémellaire [17]. Cette proportion ne dépassait pas deux fois dans notre étude. D'autre part, nous n'avons recensé dans notre étude aucun cas d'accouchement par voie basse pour le premier jumeau suivi d'une césarienne en urgence pour le deuxième jumeau alors que ce mode d'accouchement a représenté 4,34% des cas dans l’étude de Persad *et al*. [16]. L'intervalle entre l'accouchement des jumeaux était en moyenne de 6,28 minutes dans notre série. Cet intervalle n'a dépassé 30 minutes que dans 1,54% des cas dans notre étude, alors qu'il l'a été dans 5,13% pour Sarojini *et al*. [18]. Ce taux dépassait 10% dans des séries moins récentes [19, 20].

L’âge gestationnel moyen des jumeaux dans notre étude était de 37,08 semaines ce qui est similaire aux moyennes retrouvées par différents auteurs [21, 22]. Il était cependant plus élevé que celui retrouvé par Assunção *et al*. (34,40 semaines) et par Bhavana *et al*. (35,40 semaines) [15, 23]. En réalité, l’âge gestationnel moyen est inversement lié à la multiplicité. Une étude réalisée aux États-Unis a retrouvé que l’âge gestationnel moyen était de 39 semaines pour les singletons, de 35,8 semaines pour les jumeaux et de 32,5 semaines pour les triplés [24]. Minakami et Sato ont suggéré que la date estimée d'accouchement pour les grossesses gémellaires était réglée à une gestation entre 37 et 38 semaines plutôt qu’à une durée de 40 semaines [25]. Ils ont également montré dans leur étude réalisée au Japon que les taux de mortalité périnatale étaient les plus bas lorsque l’âge gestationnel était de 38 semaines pour les singletons et de 37 semaines pour les jumeaux. La prématurité est un contributeur majeur à la morbimortalité périnatale dans les grossesses gémellaires [24]. La gémellité a été responsable de 80,00% des cas de prématurité dans notre étude et le pourcentage des naissances prématurées en cas de grossesse gémellaire était de 27,69%. Ce dernier taux était supérieur à celui retrouvé par Attah *et al*. (22,30%) et inférieur à celui constaté par Tempe (36,34%) [19, 21]. Certains auteurs ont retrouvé des taux encore plus élevés. Kore *et al*. et Rizwan *et al*. ont rapporté respectivement des taux de 68,4% et 84,36% de prématurité dans les grossesses gémellaires [26, 27]. D'autre part, la prématurité constituait la morbidité périnatale la plus fréquente après l'hypotrophie chez les nouveau-nés à terme dans notre étude et elle constituait la complication la plus fréquente dans beaucoup d’études [13, 28, 29]. Dans notre étude, les jumeaux nés prématurément étaient significativement plus sujets à développer une détresse respiratoire néonatale et à être hospitalisés en période néonatale que les jumeaux à terme. La prématurité est en effet le facteur le plus important qui contribue à l'augmentation de la morbimortalité périnatale dans les grossesses multiples [30]. La rupture prématurée des membranes a compliqué 29,23% des grossesses gémellaires dans notre étude, elle est souvent associée à une infection maternofœtale et conduit fréquemment à un accouchement prématuré [31]. La prévention des complications de la prématurité passe par la prévention de l'infection et la corticothérapie anténatale. Celle-ci a été effectuée dans deux tiers des cas pour les grossesses de moins de 35 semaines de gestation. L'antibiothérapie pour la prophylaxie de l'infection à streptocoque du groupe B n'est pas réalisée de routine dans notre maternité. En outre, l'hypotrophie était la morbidité périnatale la plus courante chez les jumeaux à terme dans notre étude. Selon Sarojini *et al*., la mortalité néonatale liée à l'hypotrophie pourrait être considérablement réduite si on pouvait atteindre un poids de naissance supérieur à 1500 g chez les jumeaux et si l'on dispose d'une unité de soins intensifs et de réanimation néonatale adéquate [18]. Dans notre série, le taux de mortalité périnatale des jumeaux était de 30,8 pour mille naissances et il était 4,3 fois supérieur à celui des singletons. Qazi avait retrouvé un taux 5,2 fois supérieur dans son étude [32]. Cette différence est attribuable aux taux plus élevés de prématurité et d'hypotrophie dans les grossesses gémellaires [33].

La proportion des grossesses monochoriales était dans notre étude de 24,62%, ce qui est comparable aux fréquences retrouvées dans d'autres études [32, 34]. Dans ce sous-groupe des grossesses gémellaires monochoriales, la chorionicité a constitué un facteur pronostique défavorable. La chorionicité n'a pas significativement influencé les taux de naissances prématurées dans notre étude. L’âge gestationnel moyen des jumeaux monochoriaux était cependant inférieur d'environ une semaine dans notre étude par rapport à celui des jumeaux bichoriaux sans que cette différence ne soit significative. Dans l’étude d'Assunção *et al*., cette différence était significative et les auteurs ont montré que le nombre de jumeaux prématurés de moins de 32 semaines était 2,5 fois plus élevé dans les grossesses monochoriales par rapport aux grossesses bichoriales. L’âge gestationnel était encore plus faible en cas de complications telles que les malformations ou un syndrome transfuseur-transfusé [23]. De même, les jumeaux monochoriaux avaient un poids de naissance significativement plus faible par rapport à celui des jumeaux bichoriaux et la différence était significative dans notre étude. Blecker et Hemrika avaient obtenu des résultats similaires [35]. Le taux de discordance du poids de naissance entre les jumeaux monochoriaux était également significativement plus élevé que chez les jumeaux bichoriaux et ce même constat a été retrouvé par Hatkar et Bhide [36]. Dans notre étude, la mortalité périnatale était significativement plus élevée pour les jumeaux monochoriaux par rapport aux jumeaux bichoriaux. Des résultats similaires ont été retrouvés par Peter *et al*. et Dubé *et al*. [34, 37]. Cette issue défavorable est en partie la conséquence d'une fréquence plus élevée de la prématurité, ce qui nécessite une surveillance plus rapprochée des grossesses gémellaires monochoriales [38]. Et étant donné que le diagnostic de la chorionicité est fiable par échographie obstétricale dès 10-14 semaines de gestation comme l'avait montré Sepulveda *et al*., la détermination précoce de la chorionicité permet le diagnostic précoce des complications des grossesses gémellaires monochoriales [39]. Cette étude comble une lacune dans la littérature sur l'issue périnatale des grossesses gémellaires au Maroc. Nos résultats pourront guider l’élaboration de stratégies de prise en charge des grossesses gémellaires à l'avenir dans notre pays. Certaines limitations doivent toutefois êtres signalées. En dépit de l'implication des résidents en pédiatrie dans cette étude, il nous manquait certaines données maternelles telles que le statut tabagique, la prise de médicaments et l'utilisation des plantes pendant la grossesse. En effet, en dehors des heures de travail, il y avait uniquement un résident en pédiatrie de garde à la maternité Souissi pour faire face à un nombre élevé de naissances et ces informations n'ont pas pu être recueillies chez toutes les mères de nouveau-nés sains. Nous n'avons par conséquent pas pu intégrer ces paramètres dans notre étude. Des données néonatales étaient également manquantes puisqu'aucun autre suivi n'a été effectué après la sortie de la maternité ou après la décharge avant sept jours de vie des nouveau-nés hospitalisés. Une autre restriction était le nombre limité de grossesses gémellaire recrutées dans notre étude et qui était lié à la durée de l’étude. Afin de renforcer ces résultats, nous recommandons de conduire une étude similaire impliquant toute l’équipe pédiatrique et obstétricale pendant une plus longue période et avec un suivi de tous les nouveau-nés jusqu’à sept jours de vie.

## Conclusion

Les grossesses gémellaires constituent un facteur de risque significatif de morbimortalité périnatale par rapport aux grossesses simples et sont de ce fait des grossesses à risque. La morbimortalité périnatale est encore plus importante en cas de grossesses monochoriales par rapport aux grossesses bichoriales, faisant des grossesses gémellaires monochoriales des grossesses à risque plus élevé. La prématurité est la principale cause de l'augmentation de la morbimortalité périnatale dans les grossesses gémellaires. Elle doit être prévenue par l'utilisation de la corticothérapie anténatale de routine et la prévention des menaces d'accouchement prématuré. L'hypotrophie et la discordance du poids de naissance sont les complications les plus importantes des grossesses monochoriales. L'accent doit être mis sur une meilleure identification et un meilleur suivi des grossesses gémellaires. Le diagnostic précoce de la chorionicité et le suivi échographique périodique sont fondamentaux. Un suivi spécifique sera nécessaire en fonction de la chorionicité et de la présence éventuelle de complications maternelles ou fœtales. Tous les efforts doivent être faits dans le but de prolonger l’âge gestationnel, augmenter le poids de naissance et optimiser le mode d'accouchement. Dans des conditions optimales, un âge gestationnel cible de 37-38 semaines doit être l'objectif à atteindre avant l'accouchement pour réduire la morbimortalité périnatale des grossesses gémellaires. Nous recommandons enfin que les grossesses gémellaires compliquées soient orientées vers une maternité de niveau 3 pour un suivi étroit de ces grossesses et pour l'optimisation du moment et du mode d'accouchement ainsi que l'apport de soins néonatals de qualité.

### Etat des connaissance sur le sujet

- Les grossesses gémellaires exposent à un risque de morbimortalité périnatale plus élevé que les grossesses simples.
- Il n'existe pas à notre connaissance d’études marocaines concernant le profil périnatal des grossesses gémellaires.

### Contribution de notre étude à la connaissance

- Cette étude comble une lacune dans la littérature sur l'issue périnatale des grossesses gémellaires au Maroc.
- Nos résultats pourront aider à l’élaboration de stratégies de prise en charge des grossesses gémellaires au Maroc.
